# Cutaneous Horn Revisited: A Woman With a Verruca Vulgaris-Associated Cornu Cutaneum

**DOI:** 10.7759/cureus.19925

**Published:** 2021-11-26

**Authors:** Alexander R Kheshvadjian, Christof Erickson, Antoanella Calame, Philip R Cohen

**Affiliations:** 1 Medicine, Rutgers New Jersey Medical School, Rutgers University, Newark, USA; 2 Dermatology, Compass Dermatopathology, San Diego, USA; 3 Dermatology/Dermatopathology, Compass Dermatopathology, San Diego, USA; 4 Dermatology, Scripps Memorial Hospital La Jolla, San Diego, USA; 5 Dermatology, University of California Davis Medical Center, Sacramento, USA

**Keywords:** vulgaris, verruca, squamous cell carcinoma, seborrheic, keratosis, keratoacanthoma, horn, cutaneous, carcinoma, actinic

## Abstract

A cutaneous horn, sometimes referred to as cornu cutaneum, is a projection arising on the skin due to an overgrowth of the epidermal stratum corneum. This lesion is a clinical presentation of an underlying skin tumor. A woman with a verruca vulgaris-associated cutaneous horn is described. Cutaneous horns are often solitary and appear most commonly on the face, ears, and the dorsum of the hands of older patients. The most frequent tumors associated with cutaneous horns include actinic keratoses and seborrheic keratoses; however, cutaneous horns have also been observed overlying other benign and malignant tumors. In conclusion, a cutaneous horn is a common clinical feature; however, the diagnosis of the underlying skin lesion requires a biopsy that permits adequate microscopic evaluation of the associated tumor.

## Introduction

Cutaneous horns are described as exophytic keratotic mounds overlying the epidermis. In order for the lesion to be termed as a “horn,” the height of the lesion must be greater than at least one-half of its greatest diameter. The most common sites for cutaneous horns include the face, ears, and the dorsum of the hands of older patients [1,2]. 

Cutaneous horns can be associated with benign, premalignant, or malignant tumors. They are most frequently seen overlying an actinic keratosis; however, they are commonly associated with seborrheic keratosis and verruca vulgaris. When a cutaneous horn is observed, a biopsy is necessary to evaluate and determine the associated underlying tumor [2].

Verruca vulgaris is a human papillomavirus-associated lesion. It typically presents as a papule. Occasionally, a verruca vulgaris will develop massive hyperkeratosis and clinically present as a cutaneous horn [2]. 

A woman with a cutaneous horn-related verruca vulgaris is described. The incidence of verruca vulgaris-associated cutaneous horn and the histologic features of the lesion are reviewed. Other benign skin neoplasms, precancerous cutaneous lesions, and malignant skin tumors that have been noted to present as cutaneous horns are also discussed. 

## Case presentation

**Figure 1:**
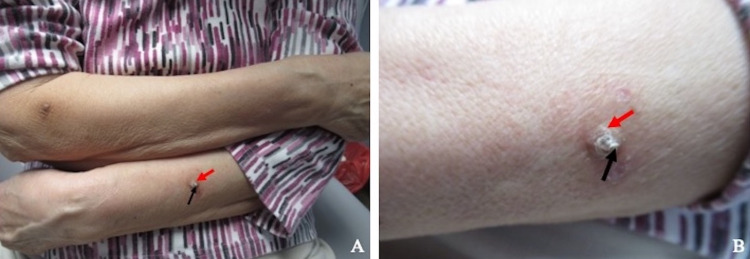
Clinical presentation of verruca vulgaris-associated cutaneous horn Distant (A) and closer (B) views of a verruca vulgaris (red arrow) associated with overlying cutaneous horn (black arrow) on the left forearm of an 81-year-old woman.

An 81-year-old woman presented for evaluation of an enlarging lesion on her left forearm of two-years duration. She had applied garlic to the lesion; however, there was no improvement. Complete cutaneous exam revealed a tan 11 x 10 x 2 millimeter keratotic nodule with a peripheral collarette and central horn on the left forearm (Figure 1). The entire lesion was removed using the shave biopsy technique. 

**Figure 2:**
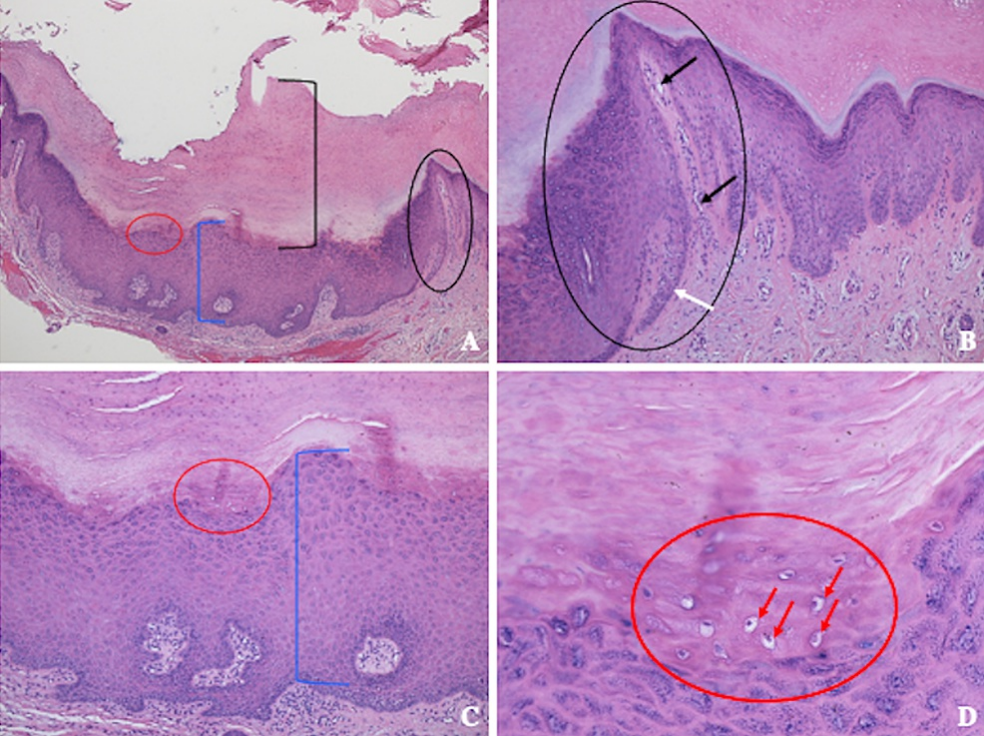
Pathologic features of a cutaneous horn associated with a verruca vulgaris Distant (A) and closer (B, C, and D) views of verruca vulgaris with an overlying cutaneous horn. There is massive thickening of the stratum corneum (black bracket) showing hyperkeratosis consisting of compact keratin with retained cell nuclei (parakeratosis) (A); these are the pathologic features of a cutaneous horn. The cup-shaped lesion shows prominent thickening of the epidermis (blue bracket demonstrating acanthosis) (A and C). There is also inward pointing of the epidermis at the lateral edge (black circle) (A and B); in this same area, there are dilated blood vessels in the dermal papillae (black arrows) and an elongated epidermal rete ridge (white arrow). In the thickened stratum granulosum (red circle) (A, C, and D), there are halo cells (red arrows showing koilocytes) (D) indicative of human papillomavirus infection; these are the pathologic features of a cup-shaped verruca vulgaris.

Microscopic examination, at low magnification, showed an epithelial tumor with a cup-shaped pattern suggestive of verruca vulgaris. There was massive hyperkeratosis, prominent acanthosis, and slight papillomatosis. At the lateral edge of the lesion, there was an inward pointing of the epidermis. A central horn-like mass of parakeratotic keratin, overlying the epidermal lesion, was indicative of a cutaneous horn. Higher magnification showed koilocytes in the upper layers of the epidermis, elongated epidermal rete ridges, and dilated blood vessels in the dermal papillae (Figure 2).

Correlation of the pathologic findings and clinical presentation established the diagnosis of cup-shaped verruca vulgaris associated with an overlying cutaneous horn. Follow-up evaluation three weeks later showed no sign of recurrence. No further treatment was required. 

## Discussion

Cutaneous horns, also known as cornu cutaneum, are keratotic growths projecting upward on the surface of the skin. They are more commonly seen in elderly populations between the ages of 60 and 80 years. They can originate from skin lesions that are either benign, precancerous, or malignant [2]. 

Cutaneous horns typically present as asymptomatic lesions; however, they can be painful. Morphologically, they appear as a hard, cone-shaped exophytic growth located in sun-exposed areas. The lesions are variable in width and height. When greater than one centimeter in height, cutaneous horns are referred to as “giant” [3]. 

Our patient had a cutaneous horn associated with verruca vulgaris. Indeed, verruca vulgaris is a frequently observed tumor underlying a cutaneous horn. Four retrospective studies showed the occurrence of verruca vulgaris-associated cutaneous horn to range from 13.1% to 30.0% (median, 16.2%). Summarizing the data, 16.5% (167 of 1013) of the cutaneous horn-related neoplasms were verruca vulgaris (Table 1) [2,4-6]. 

**Table 1:** Occurrence of verruca vulgaris-associated cutaneous horn Ref, references

Author	Year	Number of verruca vulgaris	Number of cutaneous horns in study	Percentage of verruca vulgaris	Ref
Mehregan	1965	30	100	30.0	[2]
Yu et al.	1991	100	643	15.6	[4]
Mencia-Gutierrez et al.	2004	8	48	16.7	[5]
Mantese et al.	2010	29	222	13.1	[6]
Total		167	1013	16.5	[2,4-6]

In addition to verruca vulgaris, cutaneous horns have been observed in association with other skin lesions. These include not only benign neoplasms, but also precancerous lesions such as actinic keratosis, and malignant tumors. Seborrheic keratosis is a commonly observed benign neoplasm underlying a cutaneous horn whereas keratoacanthoma, squamous cell carcinoma in situ, and squamous cell carcinoma are frequently observed cutaneous horn-related malignant neoplasms [1-6]. 

On rare occasions, giant cutaneous horns can grow several inches in length with varying protrusions resembling tree branches, which can impair hand function. A 39-year-old man with multiple hard protrusions up to 15 centimeters in length extending from the base of his dorsal hand and over the fingers has been described [7]. Another patient, a 41-year-old man, had multiple giant cutaneous horn-like lesions on both hands and feet which grew up to five centimeters in diameter and up to 21 centimeters in length [8]. Histopathological examination of both patients’ lesions showed hyperkeratosis, acanthosis, and papillomatosis, with elongated rete ridges and koilocytes, establishing the diagnosis of an underlying verruca vulgaris. Both patients had the cutaneous horns removed surgically [7,8]. 

The clinical significance of a cutaneous horn is defined by the diagnosis of the underlying tumor [9]. Therefore, a biopsy or excision that provides an adequate sampling of the tissue beneath the keratotic horn is required to establish the diagnosis of the associated lesion. Once the diagnosis is established, treatment of the underlying lesion can be performed.

The management of a verruca vulgaris-associated cutaneous horn may require no additional therapy if the lesion has been removed during the biopsy. However, if verruca vulgaris is still present clinically or the lesion extends to the margins of the biopsy specimen, treatment of the residual verruca vulgaris would be similar to that of a verruca vulgaris that was not associated with a cutaneous horn. Hence, potential therapeutic interventions could include destructive treatments (such as cryotherapy with liquid nitrogen, electrodesiccation and curettage, and laser), excision, or immunologic treatment (such as topical imiquimod or intralesional Candida antigen) [7,8]. If the cutaneous horn is not associated with verruca vulgaris, the appropriate management should be dictated by the diagnosis of the lesion beneath the horn [1,2,4-6].

## Conclusions

Cutaneous horns are keratotic epithelial lesions with an associated underlying benign, precancerous, or malignant neoplasm. We described a woman with a verruca vulgaris-related cutaneous horn. The incidence of verruca vulgaris as the tumor associated with a cutaneous horn is 16.5%. Seborrheic keratosis and cutaneous malignancies of squamous cell derivation are also commonly associated with a cutaneous horn. The observation of a cutaneous horn should prompt the clinician to secure a biopsy that provides adequate tissue to establish the diagnosis of the underlying skin tumor. 
